# Supplementary material for: Secretome Analyses Identify FKBP4 as a GBA1-Associated Protein in CSF and iPS Cells from Parkinson’s Disease Patients with GBA1 Mutations
Source: Int J Mol Sci. 2024 Jan 4;25(1):683. doi: 10.3390/ijms25010683 (PMC10779269; doi:10.3390/ijms25010683)
Supplement: Supplementary file 1 [file ijms-25-00683-s001.zip › ijms-2791938-supplementary.pdf]

Supplementary Figures

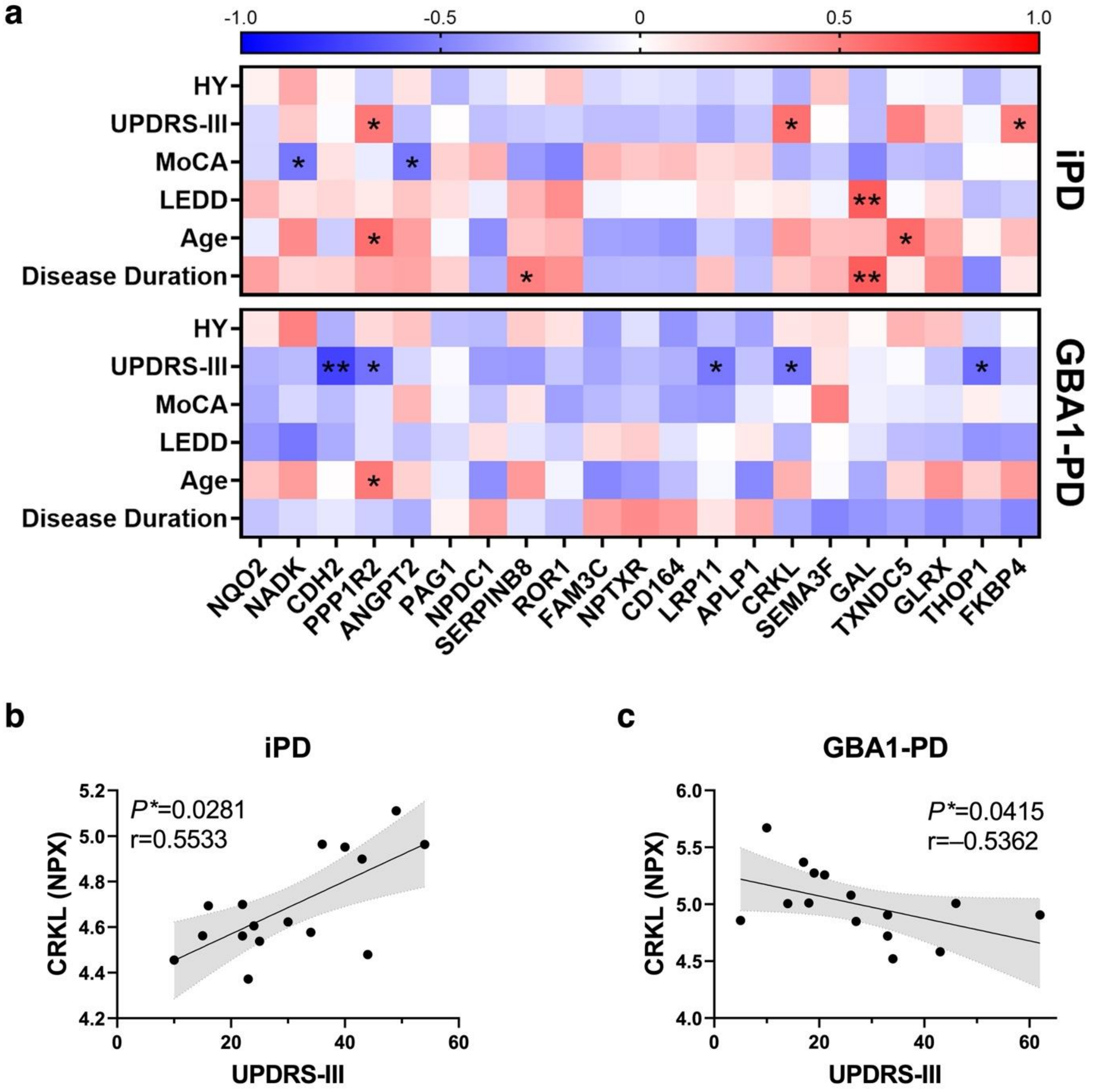

Supplementary Figure S1. Correlations of CRKL CSF levels with UPDRS-III scores. (**a**) Heatmaps showing Spearman's rank correlation coefficient between the CSF hit candidate protein levels and clinical scores of iPD and GBA1-PD. (**b**) Scatter plots showing a positive correlation between CRKL levels and UPDRS part III (UPDRS-III) scores in iPD (n = 16) and (**c**) negative correlation in GBA1-PD (n = 15). Lines represent linear regression with 95% confidence intervals. Two-tailed Spearman's rank correlation coefficient, * = $p < 0.05$, ** = $p < 0.01$

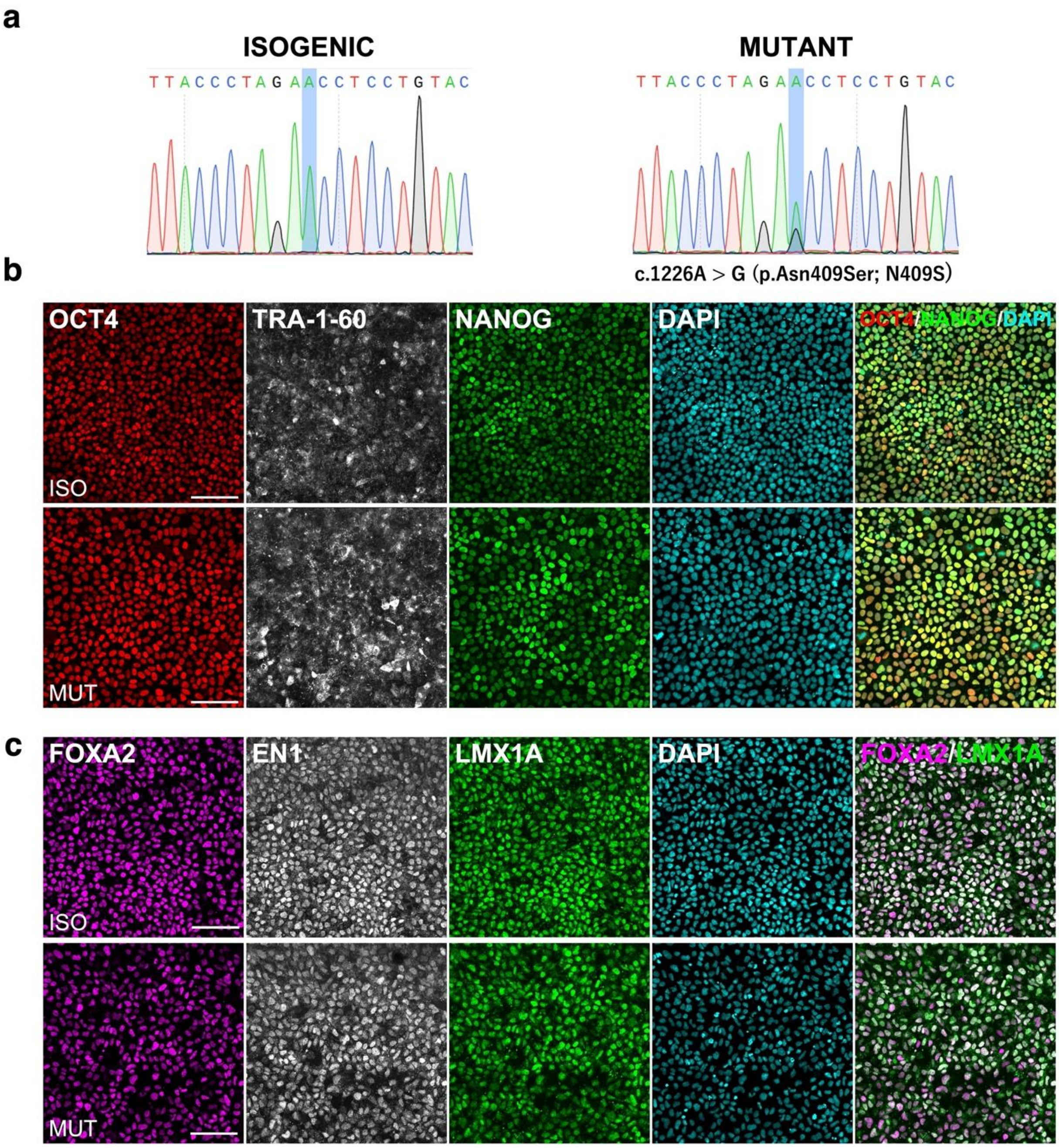

Supplementary Figure S2. Characterization of *GBA1* N409S mutant/isogenic iPSCs and mFP progenitors. (**a**) Genotype analysis on *GBA1* N409S mutant and isogenic iPSCs was conducted after 3+ passages from thawing. (**b**) Representative immunostaining of pluripotency markers (OCT4, TRA-1-60, NANOG) on undifferentiated *GBA1* N409S mutant and isogenic iPSCs. (**c**) Representative immunostaining of floor plate markers (FOXA2, EN1, LMX1A) on D11 *GBA1* N409S mutant and isogenic mFP progenitors. Scale bars, 100 μm.

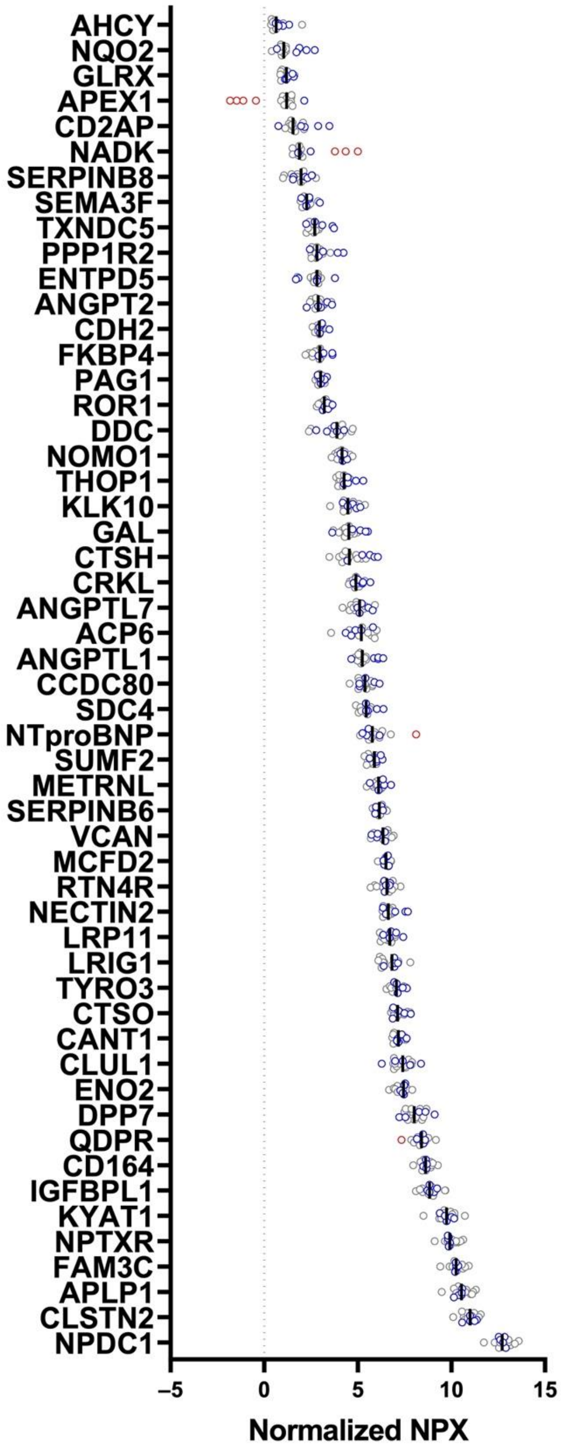

Supplementary Figure S3. Distributions of GBA1-PD CSF NPX values from two cohorts. Distributions of NPX values from 17 GBA1-PD CSF samples after normalization. Data from Cohort II (n = 5) and Cohort I (n = 12) were shown as blue and grey, respectively. Outliers colored in red were detected by the ROUT method (Q = 1%) and removed from the downstream analysis. The black bar represents the median.

Supplementary Tables

Supplementary Table S1. List of proteins included in the analysis.

Protein	Olink ID
LRIG1	OID01121
NPTXR	OID01122
AHCY	OID01123
THOP1	OID01124
CTSO	OID01125
CD164	OID01127
DDC	OID01128
ACP6	OID01129
ANGPT2	OID01132
CD2AP	OID01133
ANGPTL7	OID01134
GLRX	OID01137
ENO2	OID01138
NADK	OID01139
SERPINB8	OID01141
SERPINB6	OID01142
CCDC80	OID01144
SEMA3F	OID01147
KLK10	OID01148
ANGPTL1	OID01150
APLP1	OID01151
QDPR	OID01162
APEX1	OID01164
ENTPD5	OID01165
CLSTN2	OID01166
CLUL1	OID01168
CTSH	OID01171

Protein	Olink ID
NOMO1	OID01172
NQO2	OID01173
FAM3C	OID01175
TXNDC5	OID01176
PPP1R2	OID01177
DPP7	OID01178
LRP11	OID01179
MCFD2	OID01183
SUMF2	OID01185
CANT1	OID01186
GAL	OID01188
CDH2	OID01189
TYRO3	OID01190
CRKL	OID01191
IGFBPL1	OID01192
RTN4R	OID01193
VCAN	OID01194
NECTIN2	OID01198
FKBP4	OID01201
SDC4	OID01202
PAG1	OID01203
KYAT1	OID01204
NPDC1	OID01206
METRNL	OID01207
ROR1	OID01209
NT-proBNP	OID01214

Supplementary Table S2. List of the significantly correlated CSF proteins with clinical scores ($p < 0.05$).

iPD			
Protein	Clinical Scores	Spearman's r	*p*-value
GAL	Disease duration	0.6348	0.0074
GAL	LEDD	0.6331	0.0076
TXNDC5	Age	0.5779	0.0167
PPP1R2	Age	0.5620	0.0206
CRKL	UPDRS-III	0.5533	0.0281
ANGPT2	MoCA	−0.5335	0.0353
PPP1R2	UPDRS-III	0.5327	0.0356
NADK	MoCA	−0.5335	0.0427
SERPINB8	Disease duration	0.4975	0.0442
FKBP4	UPDRS-III	0.5092	0.0458

GBA1-PD			
Protein	Clinical Scores	Spearman's r	*p*-value
CDH2	UPDRS-III	−0.7399	0.0023
THOP1	UPDRS-III	−0.5755	0.0269
PPP1R2	Age	0.5237	0.0328
PPP1R2	UPDRS-III	−0.5559	0.0337
CRKL	UPDRS-III	−0.5362	0.0415
LRP11	UPDRS-III	−0.5273	0.0456

Supplementary Table S3. List of the significantly altered CSF proteins (q < 0.05) and the hit candidate iPSC proteins ($p < 0.05$).

Significantly altered CSF proteins (q < 0.05, with 5% FDR correction)			
Protein	Log_2FC	*p*-value	q-value
FKBP4	0.4498	0.000641	0.020880
THOP1	0.3901	0.000904	0.020880
GLRX	0.3123	0.001839	0.021499
TXNDC5	0.4063	0.001861	0.021499
GAL	0.5596	0.003327	0.025027
SEMA3F	0.3449	0.003864	0.025027
CRKL	0.2739	0.004147	0.025027
APLP1	−0.7401	0.004334	0.025027
LRP11	0.3384	0.007526	0.038634
CD164	−0.4106	0.009509	0.043627
NPTXR	−0.4939	0.010387	0.043627

Hit candidate iPSC proteins ($p < 0.05$, without correction)		
Protein	Log_2FC	*p*-value
CLUL1	−0.9152	0.005101
PPP1R2	1.348	0.007651
LRIG1	1.205	0.007696
DDC	1.986	0.010607
CCDC80	1.773	0.011934
SERPINB8	0.6518	0.013627
CRKL	1.077	0.016966
GAL	−0.6782	0.023089
FKBP4	0.8373	0.023188
ANGPTL1	1.894	0.023859
GLRX	0.6074	0.033994
NECTIN2	0.5961	0.037606
FAM3C	0.6826	0.037709
SUMF2	1.310	0.045527
THOP1	0.5371	0.048483
CANT1	0.5079	0.049715
